# Oleic acid-based nanosystems for mitigating acute respiratory distress syndrome in mice through neutrophil suppression: how the particulate size affects therapeutic efficiency

**DOI:** 10.1186/s12951-020-0583-y

**Published:** 2020-01-31

**Authors:** Huang-Ping Yu, Fu-Chao Liu, Ani Umoro, Zih-Chan Lin, Ahmed O. Elzoghby, Tsong-Long Hwang, Jia-You Fang

**Affiliations:** 1 Department of Anesthesiology, Chang Gung Memorial Hospital, Kweishan, Taoyuan Taiwan; 2 School of Medicine, College of Medicine, Chang Gung University, Kweishan, Taoyuan Taiwan; 3 Graduate Institute of Natural Products, Chang Gung University, 259 Wen-Hwa 1st Road, Kweishan, 333 Taoyuan Taiwan; 4 Graduate Institute of Biomedical Sciences, Chang Gung University, Kweishan, Taoyuan Taiwan; 5 Division of Engineering in Medicine, Department of Medicine, Brigham and Women’s Hospital, Harvard Medical School, Boston, MA USA; 6 Harvard-MIT Division of Health Sciences and Technology, Cambridge, MA USA; 7 Cancer Nanotechnology Research Laboratory (CNRL) and Department of Industrial Pharmacy, Faculty of Pharmacy, Alexandria University, Alexandria, Egypt; 8 Chinese Herbal Medicine Research Team, Healthy Aging Research Center, Chang Gung University, Kweishan, Taoyuan Taiwan; 9 Research Center for Food and Cosmetic Safety and Research Center for Chinese Herbal Medicine, Chang Gung University of Science and Technology, Kweishan, Taoyuan Taiwan; 10 Department of Chemical Engineering, Ming Chi University of Technology, New Taipei City, Taiwan

**Keywords:** Oleic acid, Lipid-based nanoparticles, Size, Anti-inflammation, Neutrophil, Acute respiratory distress syndrome

## Abstract

**Background:**

Oleic acid (OA) is reported to show anti-inflammatory activity toward activated neutrophils. It is also an important material in nanoparticles for increased stability and cellular internalization. We aimed to evaluate the anti-inflammatory activity of injectable OA-based nanoparticles for treating lung injury. Different sizes of nanocarriers were prepared to explore the effect of nanoparticulate size on inflammation inhibition.

**Results:**

The nanoparticles were fabricated with the mean diameters of 105, 153, and 225 nm. The nanocarriers were ingested by isolated human neutrophils during a 5-min period, with the smaller sizes exhibiting greater uptake. The size reduction led to the decrease of cell viability and the intracellular calcium level. The OA-loaded nanosystems dose-dependently suppressed the superoxide anion and elastase produced by the stimulated neutrophils. The inhibition level was comparable for the nanoparticles of different sizes. In the ex vivo biodistribution study, the pulmonary accumulation of nanoparticles increased following the increase of particle size. The nanocarriers were mainly excreted by the liver and bile clearance. Mice were exposed to intratracheal lipopolysaccharide (LPS) to induce acute respiratory distress syndrome (ARDS), like lung damage. The lipid-based nanocarriers mitigated myeloperoxidase (MPO) and cytokines more effectively as compared to OA solution. The larger nanoparticles displayed greater reduction on MPO, TNF-α, and IL-6 than the smaller ones. The histology confirmed the decreased pulmonary neutrophil recruitment and lung-architecture damage after intravenous administration of larger nanoparticles.

**Conclusions:**

Nanoparticulate size, an essential property governing the anti-inflammatory effect and lung-injury therapy, had different effects on activated neutrophil inhibition and in vivo therapeutic efficacy.

## Background

Oleic acid (OA) is a monounsaturated omega-9 fatty acid that is abundant in plant and animal fats. This fatty acid can have both favorable and unfavorable effects in the immune system. OA can suppress, enhance, or synergize the hyperactivity of human neutrophils depending on the experimental condition and applied dose [1]. Our previous study [2] demonstrated the effectiveness of OA for inhibiting upregulated superoxide anion and elastase in activated neutrophils. Gonçalves-de-Albuquerque et al. [3] also affirmed the usefulness of OA for decreasing sepsis mortality via reactive oxygen species (ROS) reduction. OA is a compound with an extremely high lipophilicity (*n*-octanol–water partition coefficient = 6.8). It is infeasible to formulate OA into a conventional drug vehicle, especially the injectable aqueous formulation [4]. Nanoparticulate encapsulation is an efficient approach to improving the solubility of highly lipophilic molecules while retaining injectability [5]. The other benefits of injectable nanocarriers for drug delivery include sustained release, prolonged half-life in circulation, increased drug stability, and targeting capability to a nidus [6]. Among the nanocarriers, lipid-based nanosystems show an ideal biodegradability and biocompatibility to entrap lipophilic chemicals. Our previous investigation [7] showed that OA loading in cilomilast-containing lipid nanocarriers could synergize the inhibition of psoriatic inflammation. In addition to the role as an active ingredient, OA can be a stabilizing and emulsifying agent in nanoparticles to produce highly uniform nanosystems [8–10].

The composition and size of nanocarriers is critical to governing the cellular uptake, targeting facility, and therapeutic outcome [11]. In order to maximize the therapeutic and delivery efficiency, we aimed to modulate the particulate size of OA-loaded nanocarriers for evaluating activated neutrophil inhibition, biodistribution, and anti-inflammatory therapy. The present work developed the injectable nanosystems using generally regarded as safe (GRAS-approved) materials such as OA, mineral oil, soy phosphatidylcholine (SPC), and Poloxamer 188. We successfully fabricated lipid-based nanocarriers between 105 and 225 nm by simply changing the amount of mineral oil. The primary human neutrophils were used as a cellular model to explore the anti-inflammatory activity of the lipid-based nanoparticles in this study. We utilized a lipopolysaccharide (LPS)-stimulated mouse model of acute respiratory distress syndrome (ARDS) as an in vivo disease model. ARDS is a severe form of acute lung injury (ALI) caused by infection, sepsis, collagen vascular disease, and pulmonary hemorrhage [12]. ARDS is characterized by a massive neutrophil infiltration into the lung [13]. This had led to the potential targeting of neutrophils for ARDS attenuation.

Due to the acute property of ARDS, a quickly effective medication by injection is needed to reduce the associated mortality. Lipid-based nanocarriers are suitable for pulmonary delivery because of the possibility of deep lung deposition, prolonged release, and low toxicity [14]. We assessed the biodistribution and inflammation suppression of the nanoparticles of different sizes in the ARDS mouse model. The possible influence of the particulate size on anti-inflammation was discussed in this work. The present study’s evidence leads to the rational design of anti-inflammatory lipid-based nanoformulations.

## Results

### Physicochemical properties of OA-loaded nanocarriers

**Fig. 1:**
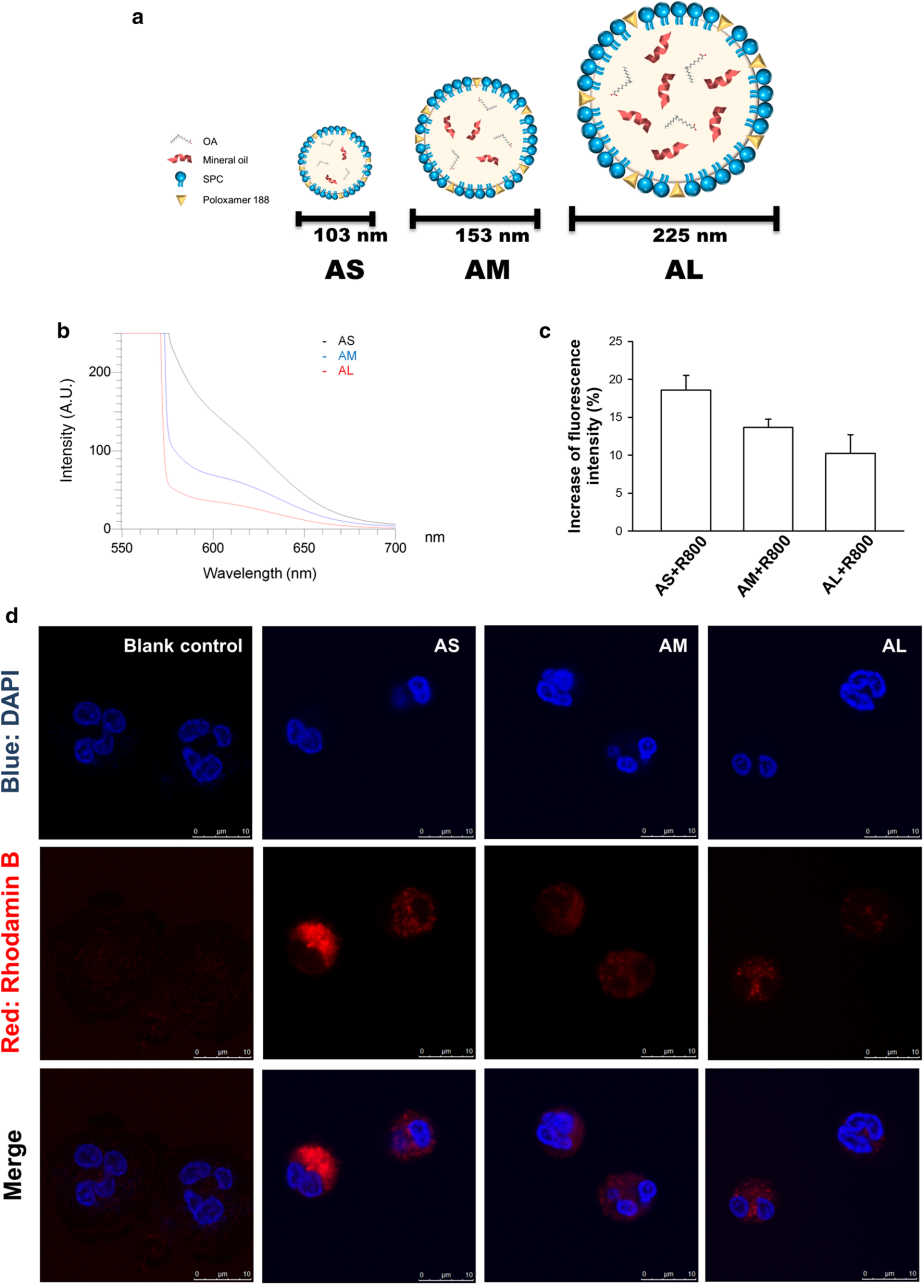
The uptake of rhodamine 800-loaded OA nanocarriers by human neutrophils. **a** The schematic exhibition of the structures of AS, AM, and AL. **b** Molecular environment (polarity) of the nanocarriers examined by Nile red fluorescence intensity. **c** The fluorescence intensity of rhodamine 800 in the neutrophils analyzed by flow cytometry. **d** Confocal microscopy of neutrophils demonstrated that rhodamine 800-loaded OA nanocarriers (red color) were internalized. Neutrophils nucleus stained (blue) were visualized by DAPI. All data represent mean ± SEM (*n *= 6)

The expected chemical configurations of three nanocarriers are illustrated in Fig. 1a. The nanoparticles were formed consisting of core lipids and emulsifier membrane. Table 1 summarizes the particulate size, polydispersity index (PDI), and zeta potential of the three nanoformulations. The average hydrodynamic diameter of the small (AS), medium (AM), and large (AL) nanoparticles determined by laser-scattering method was 105, 153, and 225 nm, respectively. This indicates that the size could be adjusted by varying the amount of mineral oil, the major material in the lipid core. The prepared nanocarriers exhibited a monodispersity. A narrow PDI (< 0.3) was achieved for AS and AM. All nanocarriers revealed a highly negative zeta potential, with the larger-sized formulations showing a greater negative surface charge. Figure 1b displays the emission spectrum of Nile red in lipid-based nanosystems. The fluorescence would be quenched in the molecular environment with lower lipophilicity. The larger-sized nanoformulations showed a weaker fluorescence, demonstrating the lesser lipophilicity as compared to the smaller size.

**Table 1:** Physiochemical properties of the lipid nanocarriers on nanoparticulate size, polydispersity index (PDI) and zeta potential

Formulation	Size (nm)	PDI	Zeta potential (mV)
AS	102.8 ± 2.8	0.25 ± 0.05	− 41.0 ± 0.7
AM	152.6 ± 1.1	0.23 ± 0.01	− 46.6 ± 0.7
AL	224.5 ± 4.4	0.33 ± 0.01	− 52.6 ± 0.1

Each value represents the mean ± SEM (*n *= 3)

### The neutrophil uptake of nanocarriers

The OA-loaded nanocarriers should interact with neutrophils for the following endocytosis and bioactivity. We investigated the effect of different sizes on nanoparticle uptake by primary human neutrophils. Flow cytometry was the tool used to offer a quantitative assessment of cellular uptake. As shown in Fig. 1c, a clear increase in the fluorescence signal was detected with decreasing particulate size. We next employed confocal microscopy to observe the uptake of nanoparticles by neutrophils as illustrated in Fig. 1d. The cells were imaged 5 min after nanoparticle exposure. The nanoparticles were quickly internalized into the neutrophils within 5 min. The fluorescence was mainly distributed in the cytoplasm without deterioration of the intact nuclei morphology. As with the use of flow cytometry, the smaller sizes showed greater uptake into the cells than the larger ones.

### Neutrophil viability

**Fig. 2:**
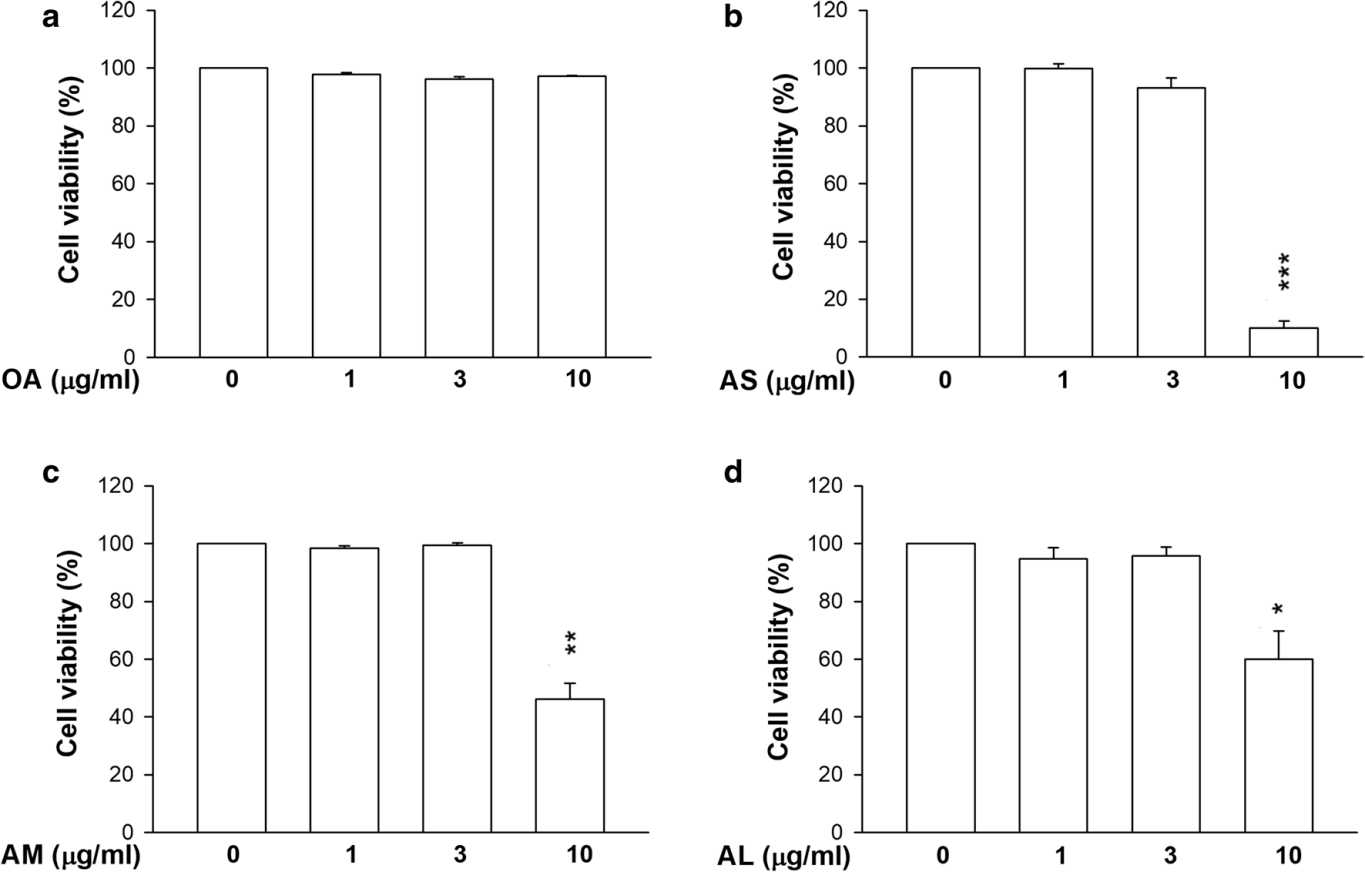
Neutrophil cytotoxicity assay by treatment of OA-loaded nanocarriers with different droplet sizes. **a** The neutrophils (6 × 10^5^ cells/ml) were treated with free OA. **b** The neutrophils (6 × 10^5^ cells/ml) were treated with AS. **c** The neutrophils (6 × 10^5^ cells/ml) were treated with AM. **d** The neutrophils (6 × 10^5^ cells/ml) were treated with AL. The cytotoxicity was measured by LDH assay. All data are expressed as the mean ± SEM (*n *= 6). *p < 0.05, **p < 0.01, ***p < 0.001 as compared to control

The cytotoxicity assay was conducted by lactate dehydrogenase (LDH) release. As depicted in Fig. 2a, free OA induced no toxicity toward the neutrophils even at a high concentration of 10 μg/ml. Nanocarrier exposure showed no cytotoxicity at OA doses of 1 and 3 μg/ml (Fig. 2b–d). The viability following treatment of nanocarriers was significantly reduced at an OA concentration of 10 μg/ml. The smaller sizes showed higher cytotoxicity against neutrophils than the larger ones at 10 μg/ml.

### Superoxide anion production and elastase release

**Fig. 3:**
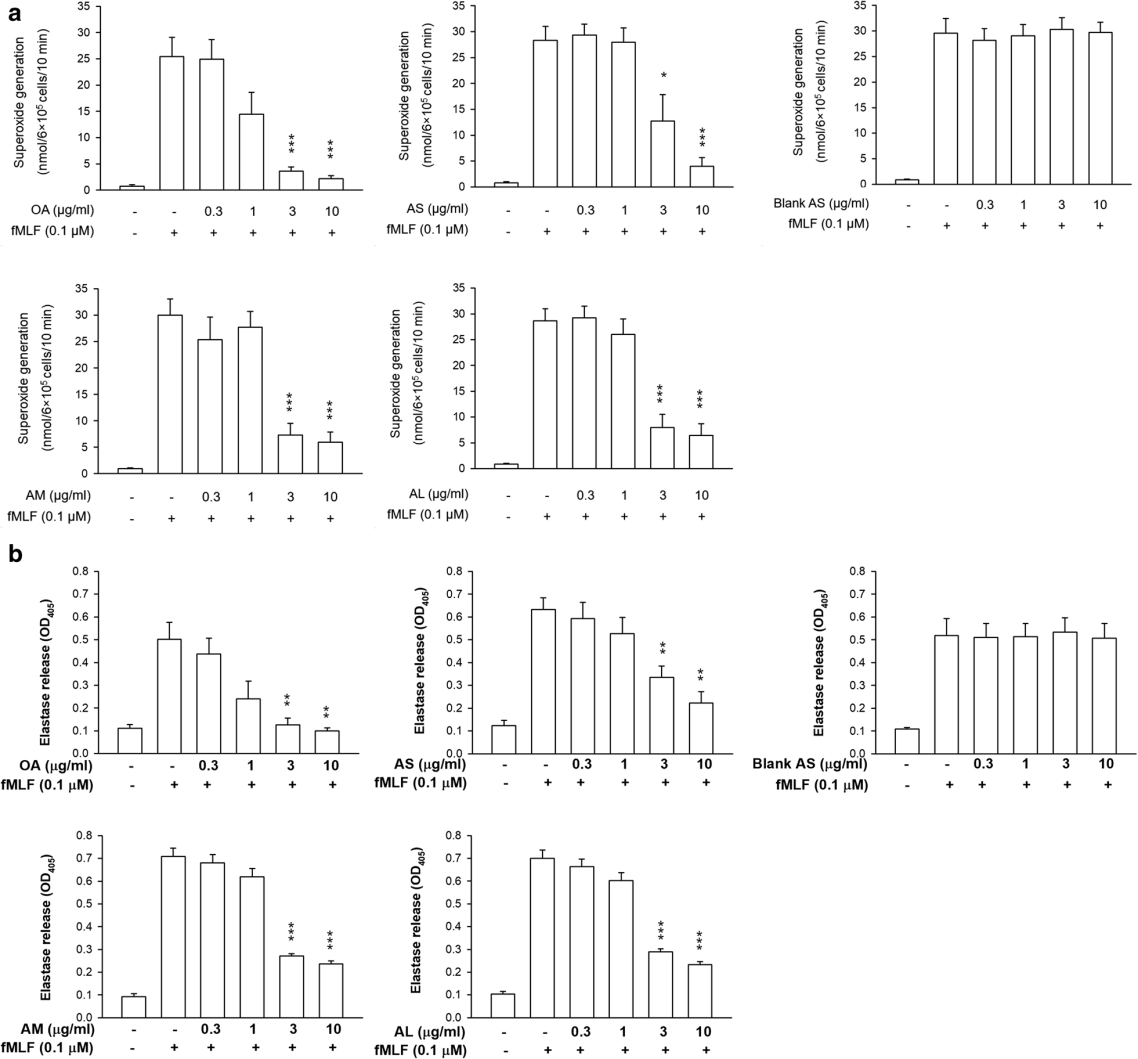
Effects of OA-loaded nanocarriers on superoxide anion release and elastase activity in fMLF-activated human neutrophils (6 × 10^5^ cells/ml). **a** The measurement of extracellular superoxide production by fMLF/cytochalasin B for 10 min. **b** Assay of absorbance at 405 nm for continuous measurement of human neutrophil elastase release. All data are expressed as the mean ± SEM (*n *= 6). *p < 0.05, **p < 0.01, ***p < 0.001 as compared to fMLF-activated group without OA intervention

Superoxide anion and elastase leakage are essential indicators of oxidative stress and degranulation in stimulated neutrophils, respectively. We tested the superoxide production and elastase release of neutrophils after the exposure of free or nanoparticulate OA. The OA concentrations of 0.3–10 μg/ml were chosen in this experiment due to the previous experience of effective neutrophil activation inhibition by free fatty acids within this concentration range [2], although the dose of 10 μg/ml caused some cytotoxicity. As shown in Fig. 3a, free OA inhibited superoxide generation in a concentration-dependent manner. Free OA at 3 μg/ml induced a nearly fivefold decrease of superoxide. The same tendency was observed in the case of AS. No superoxide suppression was found by incubation of AS in the absence of OA (blank AS). This suggests that the confinement of neutrophil activation was derived from OA but not from the other ingredients in the nanoparticles. The nanocarriers of different sizes revealed a comparable inhibition on superoxide anion. Free OA has the capacity to dose-dependently impede elastase release as shown in Fig. 3b. This effect was still detectable after encapsulation into nanoparticles. There was no difference in the elastase inhibition by nanocarriers with different diameters. Nanoparticle treatment showed less potency on elastase inhibition than the free control.

### Intracellular Ca^2+^ ([Ca^2+^]_i_) assay

**Fig. 4:**
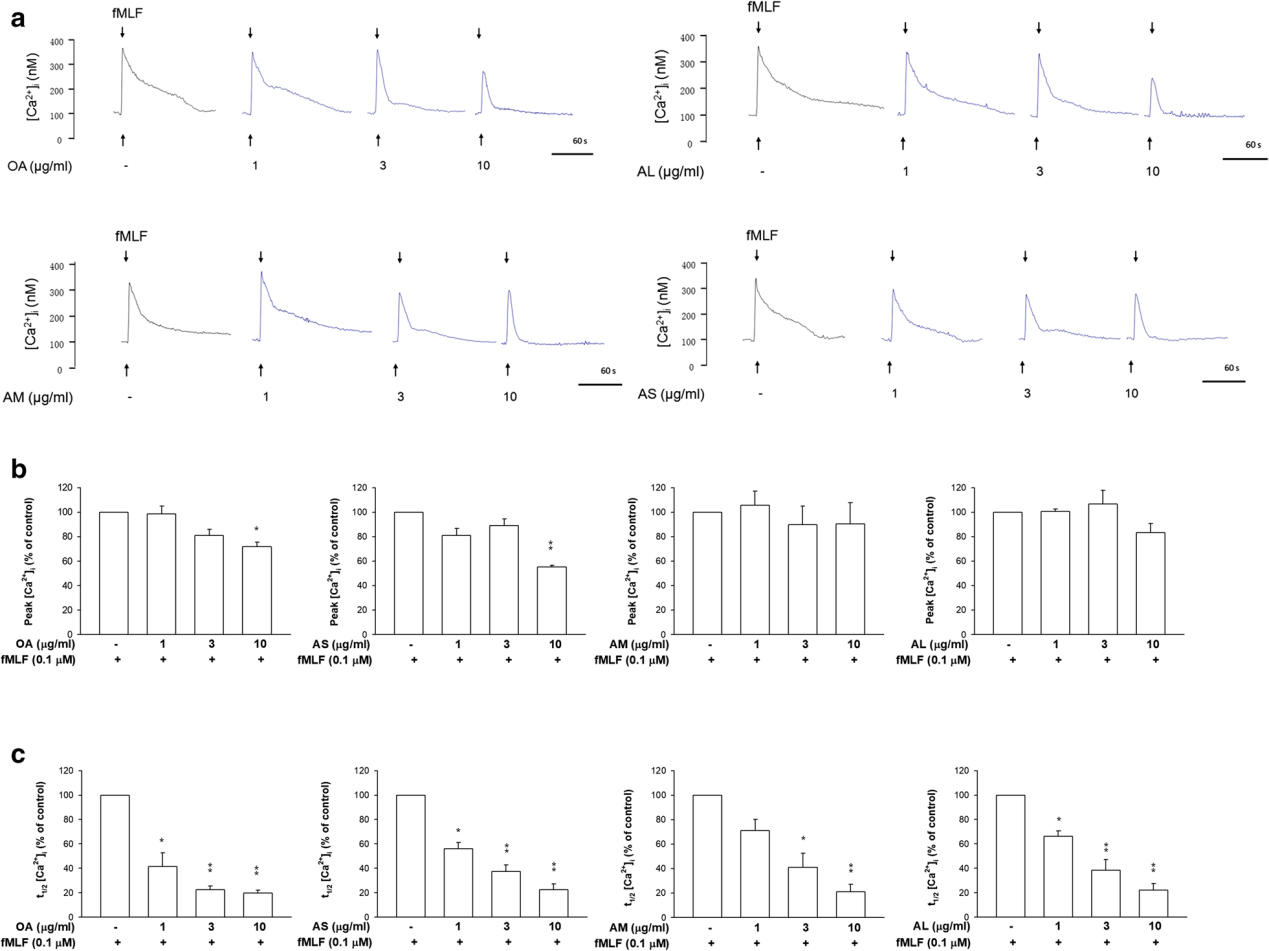
The Ca^2+^ mobilization and functional change of fMLF-activated neutrophils. **a** Fluo-3/AM-labeled neutrophils were treated with free OA and OA-loaded nanocarriers for 5 min. Next, the cells were activated by fMLF. The [Ca^2+^]_i_-time curves are shown. **b** Peak calcium concentration ([Ca^2+^]_i_) traces are shown. **c** Reduction of the time taken to decline to half of its peak values (t_1/2_) are shown. All data represent mean ± SEM (*n *= 6). *p < 0.05, **p < 0.01 as compared to fMLF-activated group without OA intervention

[Ca^2+^]_i_ presents an important role in the function of activated neutrophils. Formyl-l-methionyl-l-leucyl-l-phenylalanine (fMLF) stimulation results in a fast [Ca^2+^]_i_ increase as shown in Fig. 4a. This elevation evoked neutrophil activation. The fMLF-stimulated neutrophils were treated by free and nanoparticulate OA for observing the change of [Ca^2+^]_i_. The peak value of [Ca^2+^]_i_ in spectrofluometry shown in Fig. 4a was computed. As illustrated in Fig. 4b, peak [Ca^2+^]_i_ could be diminished in the treatments of free OA and AS at a high concentration (10 μg/ml). AM and AL failed to restrain [Ca^2+^]_i_. The time needed for [Ca^2+^]_i_ to return to half of the peak (t_1/2_) was calculated as presented in Fig. 4c. The t_1/2_ showed a reduction trend in response to free and nanoparticulate OA at all concentrations tested. The difference of t_1/2_ among all groups tested was limited.

### The formation of neutrophil extracellular traps (NETs)

**Fig. 5:**
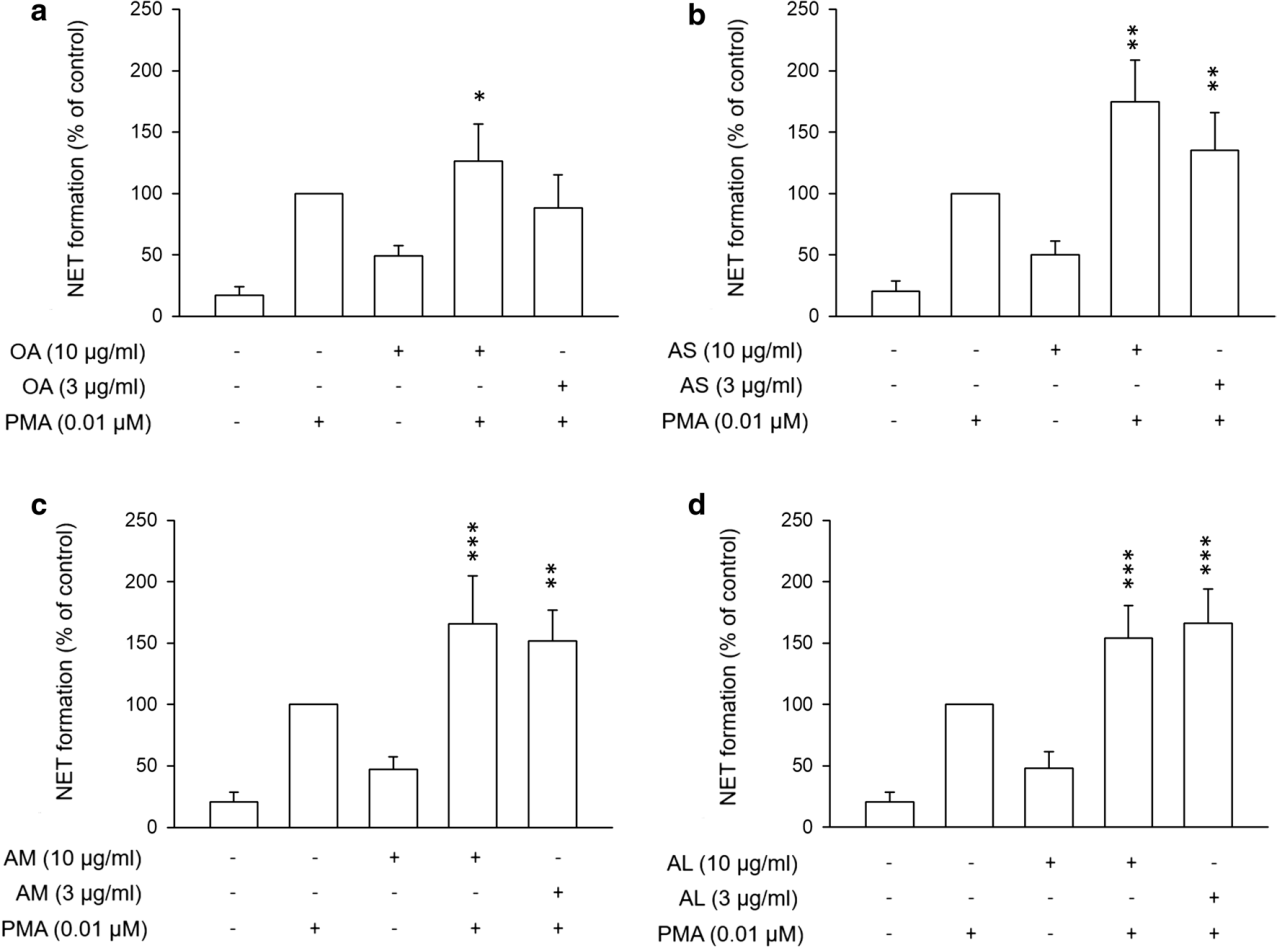
Effects of OA-loaded nanocarriers on the NET formation. **a** The PMA-activated neutrophils were treated with free OA. **b** The PMA-activated neutrophils were treated with AS. **c** The PMA-activated neutrophils were treated with AM. **d** The PMA-activated neutrophils were treated with AL. *p < 0.05, **p < 0.01, ***p < 0.001 as compared to PMA-activated group without OA intervention

Activated neutrophils undergo degranulation to release NETs. We used phorbol myristate acetate (PMA) to induce the generation of NETs. As shown in Fig. 5, PMA increased NETs by about fivefold as compared to the nontreatment control. Surprisingly, free OA at 10 μg/ml raised the amount of NETs in the absence or presence of PMA (Fig. 5a). The same tendency was detected for all nanocarriers tested (Fig. 5b–d). Since the OA dose of 10 μg/ml could cause cytotoxicity, we selected another dose (3 μg/ml) for examining the production of NETs. This low OA dose still increased NETs in the PMA-induced neutrophils.

### Biodistribution of nanocarriers

**Fig. 6:**
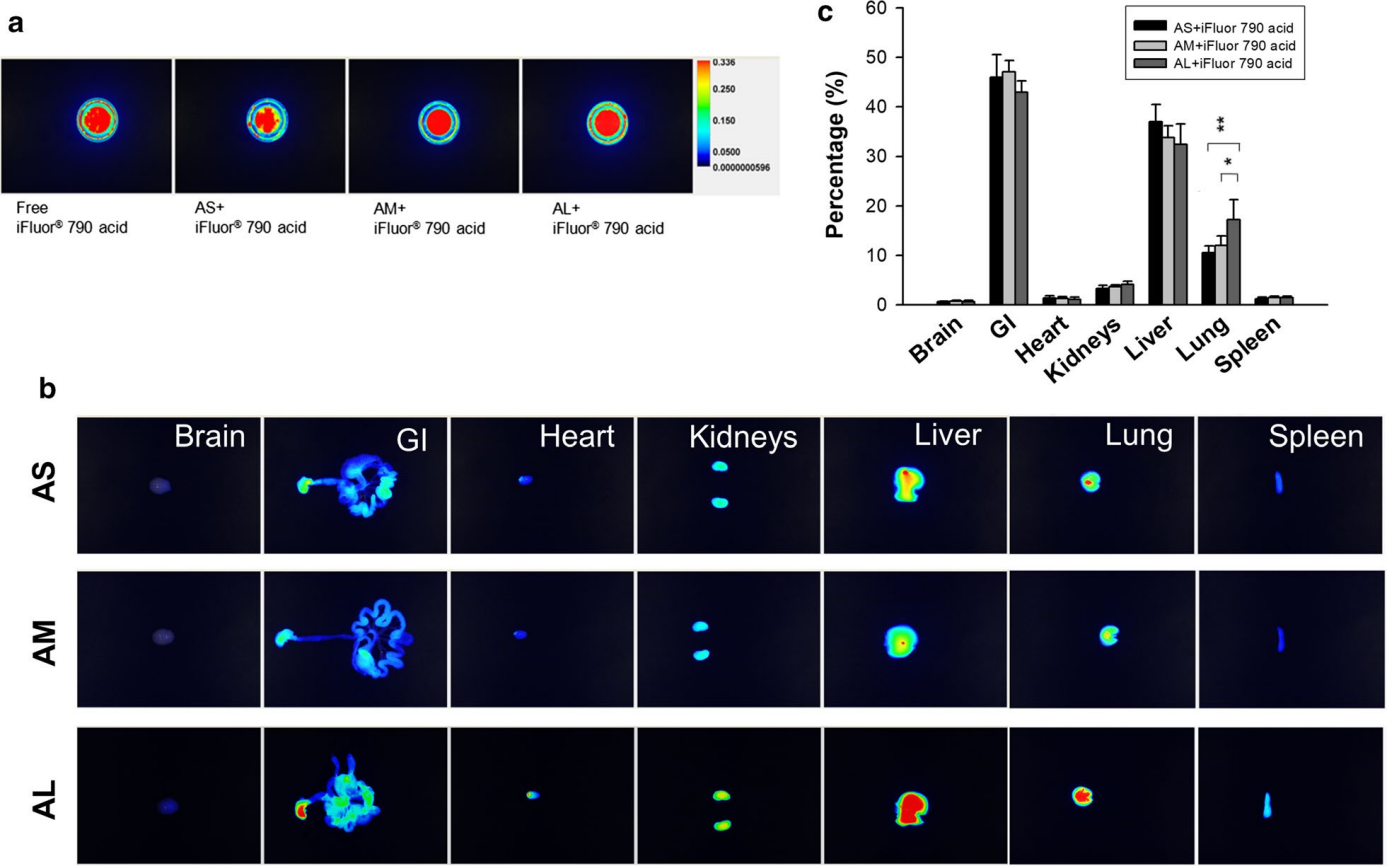
Ex vivo bioimaging of organs of the mice receiving intravenous iFlour 790 acid-loaded OA nanocarriers. **a** The NIR signal of the prepared samples of free iFlour 790 acid and iFlour 790 acid-loaded OA nanocarriers. **b** The ex vivo bioimaging of organs of representative animals. **c** The percentage (%) of NIR intensity of iFlour 790 acid in different organs analyzed by Pearl Impulse. The scale of bioimaging was calibrated by the intensity of the formulations for impartial comparison. All data represent mean ± SEM (*n *= 6). *p < 0.05, **p < 0.01 as compared to AL

The biodistribution of an active agent entrapped in nanoparticles is controlled by the size and is different from the free form. It is vital to monitor the biodistribution to understand the therapeutic efficacy. The OA-loaded nanoparticles were prepared with the addition of near infrared (NIR) dye to enable visualization using a bioimaging system. Nanoparticle encapsulation minimally changes the NIR intensity of the free dye (Fig. 6a). The three dye-containing nanocarrier samples showed a comparable NIR intensity. The nanoparticles were intravenously injected into the mice. Organs were resected at 2 h post-injection to observe NIR intensity. This time point was adequate to observe the distribution of injectable lipid-based nanoparticles in organs. Since the NIR intensity of the different nanocarrier samples might be somewhat different, we had calibrated the ex vivo organ images by the intensity of different formulations. Thus the organ images from different nanocarriers could be reasonably compared according to the same criterion. As manifested in Fig. 6b, nanoparticle accumulation is apparently high in the gastrointestinal (GI) tract, the liver, and the lung. A negligible signal was observed in the brain, indicating the minimal penetration of the nanocarriers across the blood–brain barrier. The signal in the heart and spleen was also insignificant. The larger-sized nanocarriers had a higher uptake level in the organs than the smaller ones. Figure 6c summarizes the NIR intensity percentage in the different organs. The OA nanoparticles were primarily distributed to the GI, followed by the liver and the lung. The accumulation percentages of nanosystems in the lung were 10–20%. The lung distribution was increased as size increased. Particulate size exerted an unappreciable effect on the deposition of the other organs.

### The effect of nanocarriers on ARDS treatment in mice

**Fig. 7:**
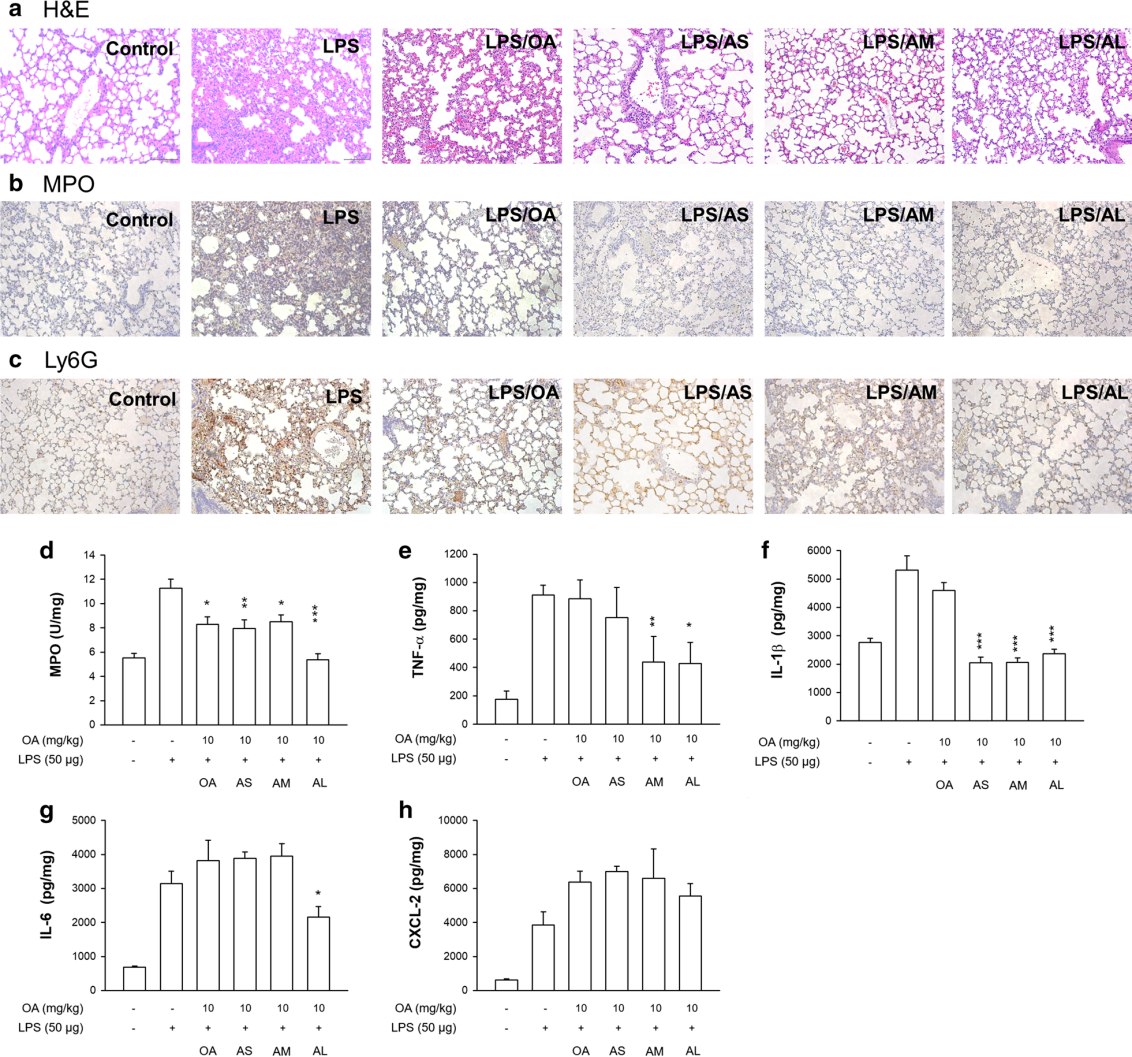
The effect of intravenous free OA and OA-loaded nanocarriers on LPS-induced lung injury in mice. **a** The lung histology (H&E staining) of LPS-challenged mice treated by free OA and OA-loaded nanocarriers. **b** The immunohistochemistry (MPO antibody staining) of LPS-challenged mice treated by free OA and OA-loaded nanocarriers. **c** The immunohistochemistry (Ly6G antibody staining) of LPS-challenged mice treated by free OA and OA-loaded nanocarriers. **d** MPO expression. **e** TNF-αexpression. **f** IL-1β expression. **g** IL-6 expression. **h** CXCL-2 expression. All data represent mean ± SEM (*n *= 6). *p < 0.05, **p < 0.01, ***p < 0.001 as compared to LPS-treated group without OA intervention

An LPS-induced mouse ARDS model was used for pharmacodynamic evaluation of nanoformulations to corroborate the biodistribution findings and therapeutic efficacy. Intravenous free OA or OA-loaded nanoparticles were administered into the ARDS-like mice. The animals were sacrificed 6 h after LPS instillation to excise the lung for the subsequent histological and biochemical assays. A 6-h period for LPS stimulation is sufficient to induce the ARDS-like syndromes. The hematoxylin and eosin (H&E) staining revealed the cause of inflammation in the LPS group (Fig. 7a), including immune cell infiltration, alveolar hemorrhage, and edema. Free OA marginally ameliorated the inflammatory signs. At the study endpoint, the inflammatory symptoms were further improved by the injectable nanocarriers. Both MPO and Ly6G are the biomarkers of neutrophil recruitment for characterizing the ADRS feature. As shown in Fig. 7b, c, a significant increase of MPO and Ly6G production in pulmonary tissue was found after LPS instillation. The neutrophil accumulation could be decreased by free and nanoparticulate OA, with the nanoparticles displaying better mitigation. The larger nanoparticles showed higher neutrophil inhibition than the smaller ones. To further confirm the histology result, we quantified the MPO amount by ELISA analysis. As shown in Fig. 7d, MPO decreased in response to the administration of free and nanoparticulate OA. The highest MPO inhibition was observed in the AL group.

We also quantified the cytokine levels in the lung to explore nanoparticle activity on proinflammatory mediators. In the case of TNF-α (Fig. 7e), the prophylactic administration of AM and AL resulted in a significantly lower expression as compared to the LPS group. This effect was not found in the groups of free OA and AS. IL-1β expression exhibited no marked inhibition by free OA (Fig. 7f). Treatment of nanocarriers significantly reduced IL-1β, with similar inhibition for the three nanoformulations. In the case of IL-6 (Fig. 7g), only AL significantly reduced its expression in the LPS-treated lung. The chemokine CXCL-2 was determined as depicted in Fig. 7h. Although the OA-loaded nanosystems clearly inhibited cytokine production in the animal model, an increase of CXCL-2 expression was achieved when administering free or nanoparticulate OA to LPS-treated mice.

## Discussion

In this study, we reported the effect of OA-loaded lipid-based nanoparticles on anti-inflammatory potency against neutrophils and the ability to mitigate ARDS in a mouse model. Our ability to control the nanoparticulate size made it possible to elucidate how the specific sizes affected anti-inflammatory efficacy. Our major findings were that the smaller sizes showed greater internalization and cytotoxicity in neutrophils. Nanoparticulate OA inhibited the superoxide and elastase of activated neutrophils with less potency compared to the free control. However, injectable OA-loaded nanoparticles demonstrated greater mitigation on lung injury in vivo than free OA. The larger-sized nanocarriers were beneficial to ameliorate ARDS with greater efficiency. There are limited drugs for treating ARDS-induced inflammation. Current data on the use of steroids in ARDS indicate no definitive evidence of reducing mortality [15]. OA-loaded nanosystems may provide an opportunity to treat ARDS in the future.

Previous study [11] demonstrated that the particulate diameters ranging between 10 and 200 nm are most relevant for physical and biochemical targeting to specific tissues. Our nanoparticles fell within this range. OA was expected to remain in the lipid core of the nanoparticles because of its high lipophilicity capable of mixing with mineral oil, the main ingredient of the lipid matrix. Nevertheless, the emulsifier nature of OA might lead to the possible intercalation of some OA molecules in the emulsifier layer of the particulate surface. The carboxyl moiety of OA is responsible for negative zeta potential since this moiety is deprotonated in the physiological environment [4]. We found that the blank AS without OA displayed a negative surface charge of − 35.2 mV, which was less than that of OA-loaded AS (− 41.0 mV). This confirmed the coating of some OA on the nanoparticulate shell. Zeta potential serves as a predominant factor to offer electrostatic repulsion among the charged nanoparticles for storage stability [16]. The high zeta potential of our nanosystems signified a great repulsion for increased stability. We developed the nanocarriers with negative surface charges with the aim of injectable anionic nanoparticles being favorable for longer circulation, leading to the possibility of enhanced distribution to the target site [17]. The anionic nanoparticles are also well tolerated in the lung whereas the cationic nanoparticles are detrimental to pulmonary function [18]. Mucin in the mucus layer of the lung is negatively charged. Mucin can capture cationic nanoparticles to retard the penetration into the alveolar space [19]. The negatively charged nanoparticles facilely transport across the mucus barrier into the alveolar space. The various sizes of OA-loaded nanoparticles are expected to penetrate through the mucus layer with a pore size range of 60–300 nm [20].

The OA nanoparticles were quickly internalized into the neutrophil cytoplasm. The surface functionalization of the nanocarriers is key to their reaction to cellular uptake. OA as the nanoparticulate stabilizer increases the probability of insertion into the cells [21]. OA interaction with membrane phospholipid bilayers can increase and disturb cellular membrane fluidity to increase invagination [1, 22]. This may be one of the reasons for the cytotoxicity induced by OA nanoparticles at a high concentration. The higher surface-to-mass ratio of AS than AM and AL for higher frequency of neutrophil interaction may contribute to the greater cytotoxicity at 10 μg/ml. Previous study [23] also suggests the enhancement of the phagocytic capacity of neutrophils by OA. Lipid-based nanocarriers could be recognized as the soft but not rigid nanoparticles. The soft nanoparticles are facilely deformed by the cells for phagocytosis although the negative charge of nanoparticulate surface might retard the interaction between cell membrane and nanoparticles. This internalization process is less energetically favorable [24]. Nanoparticulate size is a physicochemical factor to influence immune cell uptake [25]. It is generally acknowledged that the increased nanoparticulate size correlates with increased uptake by phagocytic cells [26]. This was not the case in the present study. We showed an increased neutrophil ingestion with decreased size of OA-loaded nanoparticles. The size range of our nanocarriers was 105–225 nm. The previous studies that concluded that increased size resulted in increased uptake usually used the particulate size of ≥ 300 nm as the representative of larger particles, e.g. the microscale versus nanoscale [25] and 500 nm versus 150 nm [27]. Wang et al. [28] also demonstrated the inefficient phagocytosis of nanoparticles of < 100 nm by phagocytic macrophages. Of course, the different experimental conditions and nanoparticle types might be responsible for the different results. The size range of OA-loaded lipid-based nanoparticles might display a unique property for neutrophil uptake.

Some investigations support the inverse correlation between particle size and neutrophil uptake. Kelley et al. [29] demonstrated the increased human neutrophil uptake following the size reduction of polystyrene nanoparticles. Gifford et al. [30] suggested a facile uptake of iron oxide nanoparticles with a mean diameter of 110 nm by leukocytes. The increased total surface area-to-mass ratio of the smaller sizes has promoted the opportunity to interact with cells. The smaller-sized nanoparticles often need less driving energy in the internalization procedure. The role of the surface charge and the molecular environment on neutrophil uptake cannot be ignored. The greater zeta potential causes more hydrophilicity of the nanoparticles, decreasing the cellular uptake through a stealth effect [31]. The negative surface charge and hydrophilicity of OA nanocarriers increased following the size increase, leading to less internalization. Basically, the nanoparticles would fuse with the lysosomes after invagination into the cells. The lysosomes degraded the nanoparticles to release active ingredients for revealing the bioactivity.

[Ca^2+^]_i_ is a second messenger contributing to neutrophil activation. It is a potential target for anti-inflammation. Our results showed that AS but not AM and AL significantly impeded fMLF-induced Ca^2+^ influx amplification. This could be due to neutrophils’ facile uptake of AS. All nanoformulations hastened the speed of [Ca^2+^]_i_ decline. The ability of OA to inhibit Ca^2+^ influx has been proved in neutrophils and T lymphocytes [2, 7, 32]. The augmentation of [Ca^2+^]_i_ results in neutrophil activation, producing oxidative stress and degranulation. Excess superoxide production by activated neutrophils contributes to lung tissue damage in ARDS via the release of cytokines and chemokines [13]. Elastase also holds an apparent capacity for the pathogenesis of ARDS [33]. Increased superoxide anion production and elastase release by activated neutrophils were abrogated in neutrophils internalizing OA nanoparticles. It should be noted that the inhibitory effect of the lipid-based nanocarriers at a high OA dose (10 μg/ml) was partly mediated via the cytotoxic result. No significant difference in this inhibition was detected for the nanosystems of different sizes. This suggests that although different particulate sizes showed different levels of neutrophil uptake, this effect did not influence the following alleviation of oxidative stress and degranulation. However, the cellular uptake level did affect the cytotoxicity and peak [Ca^2+^]_i_.

NETosis is a process of neutrophil degranulation in response to microbial invasion to release elastase, MPO, histones, and extracellular DNA fibers [34]. The creation of NETs is associated with ARDS severity [35]. Although OA nanoparticles could inhibit the release of elastase, NETs were augmented by this treatment. Some nanoparticles act directly on the neutrophil membrane to activate the generation of NETs. These include lipid-based, silver, polystyrene, and graphene oxide nanoparticles [36–39]. A similar effect might be found in our case. As already mentioned, OA shows controversial data involving inflammation through different mechanisms. Further investigation is necessary to elucidate the detailed mechanisms related to nanoparticle-induced NETosis. It is generally recognized that NETosis is a unique type of neutrophil death via the NADPH-oxidase (Nox)-dependent pathway [40]. Previous evidence [41] expressed that OA only induces Nox-dependent NETosis in human neutrophils. This explains the enhanced NET formation and cell death by the nanosystems with an OA dose at 10 μg/ml.

We aimed to deliver OA-loaded nanocarriers to the lung to treat ARDS. A rationale for nanoformulation design to achieve this intention was the incorporation of SPC and Poloxamer 188 as the emulsifiers. The activated neutrophils migrate to the alveolar space in ARDS development [33], promoting a large number of proinflammatory mediators. The nanoparticles should penetrate across the alveolar epithelium to reach the neutrophils. The epithelium is covered with the lining layer containing pulmonary surfactants. The main components of pulmonary surfactants are phospholipids, neutral lipids, and proteins. These surfactants can be used as efficient carriers associated with nanoparticles for delivery into the airspace [42, 43]. Our previous study [44] showed that the nanovesicles with abundant SPC were beneficial for interacting with pulmonary surfactants for lung targeting. The nanoparticles with a negative surface charge generally have a higher biodistribution than those with a positive charge [45]. The bioimaging assay displayed a broad organ distribution by intravenous OA nanocarriers. A differential response in biodistribution was detected with the different nanocarriers, with larger nanoparticles representing a more-extensive biodistribution in the peripheral organs. This can account for the longer half-life of the smaller nanoparticles in circulation after intravenous injection [26, 45]. Because of the soft and deformable characters of lipid-based nanoparticles, the larger nanoparticles such as AL facilely transported across the biological barriers such as the capillary wall.

The lung accumulation of the OA nanoparticles was size-dependent, with the larger sizes exhibiting greater uptake. The nanoparticles even with a large diameter can enter the lung tissue since the pulmonary epithelium in ARDS reveals an elevated microvascular permeability [46]. Dysfunction of the epithelial barrier facilitates the diffusion of macromolecules and nanoparticles into the alveolar space. It is well known that the lung can act as the first mechanical filter in the circulation. The lung vasculature has an extensive vascular network with 30% of the total endothelial surface. This unique characteristic enables larger nanoparticles to deposit in the lung [45, 47]. Another possibility for greater lung accumulation of larger OA-loaded nanocarriers could be that hydrophilic nanoparticles such as AL tend to rapidly penetrate across pulmonary surfactant film [48]. The plasma proteins easily adsorbed onto the intravenous nanoparticle surface to form a protein corona. This opsonization facilitated the recognition of nanoparticles by a mononuclear phagocyte system and was then engulfed by a reticuloendothelial system such as the liver, spleen, and lung. Previous investigations [47, 49] inferred that the nanoparticles between 100 and 200 nm were largely stored in the liver, whereas the nanoparticles of < 50 nm were mainly delivered to the spleen. Our data fitted this criterion, demonstrating that the OA nanocarriers showed a notable liver accumulation but a low spleen uptake. The high level of OA nanoparticles in the GI may suggest the large excretion through the biliary system from the liver to the GI tract. It is possible that nanoparticles deliver epithelial cells and hepatocytes into the bile via the bile duct [50].

MPO and cytokines in ARDS-like pulmonary tissue were analyzed to examine the anti-inflammatory effect of OA nanoparticles. MPO expressed in neutrophil granules is a major mediator of lung injury. Neutrophils also secrete some cytokines such as TNF-α, IL-1β, and IL-6 to deteriorate ARDS [51]. An attenuation of ARDS by nanoparticulate OA was found, based on the reduction of MPO and cytokines. Histological observation also confirmed a suppression of neutrophil infiltration in mice receiving OA nanoparticles. Neutrophils could be the predominant cells affected. The larger nanoparticles demonstrated greater improvement according to the data of MPO, TNF-α, and IL-6. This result correlated well with the biodistribution in the lung. Though the larger nanoparticles exhibited less capability for neutrophil uptake, the superoxide and elastase inhibition was comparable to that of the smaller sizes. The ARDS mitigation was far superior with nanocarriers than with free OA. By using nanoparticles, a robust increase of OA in the pulmonary tissue could be accomplished. Because of the extremely lipophilic feature, OA was tightly loaded in the lipid matrix with minimal leakage over an extended period, increasing the stability to prevent enzymatic attack. OA encapsulated in lipid-based nanoparticles safely delivered into the lung for neutrophil internalization, resulting in anti-inflammatory action. Free OA possibly even damaged the alveolar endothelium to evoke pulmonary capillary permeability, causing lung injury [52].

ARDS is highly associated with the activation of neutrophils. Our data verified that the inhibition of neutrophil stimulation lessened the signs of pulmonary inflammation. Although this study focused on the role of neutrophils in lung injury, the other cells related to ARDS cannot be overlooked. Lung injury involves a mixture of cells with origins of neutrophils, alveolar macrophages, dendritic cells, and T lymphocytes [53]. Cytokines can be produced by different immune cells. For instance, IL-6 is a key proinflammatory mediator secreted by neutrophils, macrophages, and T cells to induce an inflammatory cascade in ARDS [51]. Alveolar macrophages are important phagocytic cells in the lung for nanoparticle internalization. Though the larger OA nanoparticles showed less uptake by neutrophils, the larger sizes present a great opportunity to be endocytosed by macrophages [26]. Fromen et al. [18] also indicated that the nanoparticles with a high negative charge are beneficial for internalization by alveolar macrophages. Our results demonstrated the higher negative zeta potential of AL compared to that of AS and AM. NETs appear in the pulmonary microvasculature of ALI patients [54]. However, NETs can be phagocytosed and cleared by macrophages [55]. Although OA nanoparticles elicited the NET creation, this negative effect might be absent in the in vivo ARDS model due to the participation of the macrophages.

Another concern is the CXCL-2 upregulation by OA. This suggests the mechanisms other than cytokine inhibition mediated neutrophil chemoattraction. OA is proved to increase chemokines CXCL-8 and MIP-1α in lung tissue [56]. Oral OA induces CXCL-3 release and neutrophil-endothelium interaction in the air pouch [57]. The increase of CXCL-2 did not affect the overall improvement of the ARDS-like lesion by OA-loaded nanoparticles. There are some limitations in the present study. The mouse neutrophils behave differently from human neutrophils. This can cause difficulty in directly linking or correlating the in vitro results with the in vivo profiles. According to previous and present investigations, OA treatment demonstrated both advantageous and detrimental impacts on pulmonary tissue. The independent studies are hard to compare due to the variation in experimental setups such as nanoparticle types, cell models, animal models, and the administered doses. Although our nanoformulations were validated as being useful for inhibiting pulmonary inflammation, whether this effect is still maintained with different doses and clinical status remains uncertain. Further study is needed to clarify these questions.

## Conclusions

OA-based nanoparticles have attracted interest as the anti-inflammatory nanosystems against neutrophil stimulation. In the present study, we examined how the particulate size of OA nanocarriers influences inflammation suppression and the therapeutic efficiency on lung injury. The results indicated that neutrophil uptake was strongly size-dependent. Increased nanoparticle ingestion by neutrophils was observed following the decrease in size, leading to the lower cell viability and [Ca^2+^]_i_ peak. However, the constrained superoxide anion production and elastase release by nanoparticles were not affected by the size. The nanocarriers could specifically deliver to the pulmonary tissue to treat ARDS-like inflammation in mice. The greater particle diameter demonstrated superior accumulation in the lung. Compared to the smaller-sized nanoparticles, the larger sizes significantly improved the therapeutic effect against ARDS and reduced neutrophilic infiltration in the lung. The nanosystems with larger size (AL) are a better choice for ARDS treatment than the other nanoformulations because of the greater deposition in lung tissue for mitigating lung injury signs, whereas the inhibition of superoxide and elastase in stimulated neutrophils was comparable for all nanosystems. Herein, we suggest how the anti-inflammatory activity, biodistribution, and lung-injury treatment can be modulated using nanoparticle-size modification. The injectable OA-loaded nanoformulations used in this investigation can serve as an effective delivery system for ARDS therapy.

## Methods

### Fabrication of lipid-based nanocarriers

Three lipid-based nanosystems with different diameters were produced by the emulsification method. The lipid phase consisted of OA (1% of the final product, w/v), SPC (1.5%), and mineral oil. The percentage of mineral oil was 1.5%, 5%, and 10% for development of the final products with small (AS), medium (AM), and large (AL) size, respectively. The aqueous phase consisted of Poloxamer 188 (1.5%) and water. Both phases were heated separately at 85 °C for 20 min. The water phase was then added in drop form into the lipid phase via high-shear homogenization at 12,000 rpm for 20 min, followed by agitation through a probe-type sonicator (35 W) for 20 min. The nanocarriers were used in the experiments after cooling to room temperature.

### The size and zeta potential of the nanocarriers

The average diameter, PDI, and zeta potential of the OA-loaded nanoparticles were measured by a laser-scattering procedure (Nano ZS90, Malvern). The nanoparticles were diluted 100-fold with water before measurement.

### Molecular environment of the nanocarriers

The degree of lipophilicity of the nanoparticles was determined by fluorescence spectrophotometry based on the solvatochromism of Nile red [58]. Nile red (1 × 10^−3^ mg/ml) was incorporated in the lipid phase of the nanosystems. The emission spectra of dye-containing nanosystems were scanned from 550 to 700 nm. The excitation wavelength was set at 546 nm.

### Human neutrophil purification

The protocol for this purification was approved by the Institutional Review Board of Chang Gung Memorial Hospital. All subjects (20–30 years old) had signed an informed consent. The blood was collected by venipuncture. The neutrophils were isolated using dextran sedimentation before centrifugation in a Ficoll-Hypaque gradient as previously described [59].

### The neutrophil uptake of nanocarriers

The lipid-based nanoparticles were labeled with rhodamine 800 (0.1 mg/ml) as a dye to visualize neutrophil uptake (1.8 × 10^7^ cells/ml). The nanoparticles were treated with the cells for 5 min. The degree of uptake was quantified by measuring the dye fluorescence in flow cytometry. The nuclei were stained by 4′,6-diamidino-2-phenylindole (DAPI). The nanoparticle ingestion was observed under confocal microscopy (TCS SP2, Leica).

### Neutrophil viability

The neutrophil survival after nanoparticle treatment was measured by LDH release. LDH was analyzed using a commercial kit (CytoTox 96, Promega). The cells (6 × 10^5^ cells/ml) were equilibrated at 37 °C for 2 min. Subsequently, the nanocarriers with OA (1–10 μg/ml) were added into neutrophil suspension for 15 min. The OA doses used in this study were 1, 3, and 10 μg/ml. The total LDH leakage was detected after the treatment by Triton X-100.

### Superoxide anion production

We used the superoxide dismutase-inhibitable decrease of ferricytochrome *c* to detect superoxide production [59]. At first, neutrophils (6 × 10^5^ cells/ml) were equilibrated for 2 min after supplementation with ferricytochrome *c* (0.5 mg/ml) and CaCl_2_ (1 mM). Then, the nanoformulations were added to the cell suspension for 5 min. The OA doses tested were 0.3, 1, 3, and 10 μg/ml. The cells were activated by fMLF at 100 nM for 10 min. The absorbance with the reduction of ferricytochrome *c* at 550 nm was quantified by a UV/visible spectrophotometer.

### Elastase release

Meo-Suc-Ala–Ala-Pro-Val-*p*-nitroanide was employed as the substrate of elastase for detecting elastase release [60]. After the incorporation with the substrate (100 μM), the cells were equilibrated for 2 min and then treated with the nanoformulations for 5 min. We used the OA dose of 0.3, 1, 3, or 10 μg/ml for testing elastase inhibition. The neutrophils were activated by 100-nM fMLP. Absorbance at 405 nm was obtained by a UV/visible spectrophotometer.

### Intracellular Ca^2+^ ([Ca^2+^]_i_) assay

Furo-3/AM (2 μM) was used to treat the neutrophils (3 × 10^6^ cells/ml) at 35 °C for 45 min, followed by centrifugation and resuspension in Hank’s balanced salt solution with CaCl_2_ (1 mM). The cells were exposed with nanosystems for 5 min at OA dose of 1, 3, or 10 μg/ml. The [Ca^2+^]_i_ in response to fMLF was detected using a fluorescence spectrophotometer with the excitation and emission wavelength at 488 nm and 520 nm, respectively.

### The formation of neutrophil extracellular traps (NETs)

The isolated neutrophils (1 × 10^6^ cells/ml) were incubated with nanocarriers at OA concentration of 3 or 10 μg/ml for 10 min and then stimulated with PMA at 10 nM for 3 h. SYTO Green nucleic acid stain (2.5 μM) was added to the cell suspension for 15 min. The fluorescence intensity was quantified at 485 (excitation) and 535 (emission) nm, respectively [61].

### Animals

Male C57BL/6 mice (20–25 g) acquired from the National Laboratory Animal Center (Taipei, Taiwan) were used. All study procedures were conducted in accordance with the protocols approved by the Institutional Animal Care and Use Committee of Chang Gung University.

### Biodistribution of nanocarriers

We employed an NIR bioimaging system (Pearl Impulse, Li-Cor) to monitor the nanoparticle biodistribution. iFluor 790 acid (0.08%) as the NIR dye was incorporated into the nanocarriers. The nanosystems (2 ml/kg) were intravenously injected into the tail vein of the anesthetized mice. The mice were sacrificed after 2 h. The organs were excised to monitor the NIR signal using the Pearl Impulse bioimaging system.

### LPS-induced ARDS

The mice with ARDS-like signs were divided into five groups: ARDS without therapy and ARDS treated with intravenous free OA, AS, AM, or AL. An intratracheal challenge of LPS was carried out as described before [62]. In brief, we injected free OA or OA-loaded nanocarriers into the mice. LPS at 8 mg/kg was administered to the animals after a 30-min injection. The mice were sacrificed 6 h after LPS stimulation. The pulmonary tissue was excised for histological observation and ELISA analysis.

### Histological observation

The lung specimen was added into formaldehyde (10%) and embedded in paraffin. The samples were cut to a 3-μm thickness for H&E staining. For the immunohistochemistry, the slices were incubated with anti-MPO or anti-Ly6G antibody for 1 h. Subsequently, the biotinylated donkey anti-goat IgG was used to treat the samples for 20 min. A light microscope was used to visualize the slices.

### ELISA analysis

MPO activity in the lung tissue was measured by the colorimetric assay of combined *o*-dianisidine HCl and H_2_O_2_ as described previously [63]. Cytokines and chemokines in the pulmonary tissue were quantified by ELISA. The tissue was extracted with buffer containing complete protease inhibitors under homogenization (MagNA Lyser, Roche). The homogenate was centrifuged at 11,500×*g* for 10 min. The supernatant was taken to measure TNF-α, IL-1β, IL-6, and CXCL-2 employing commercial kits (BioLegend).

### Statistical assay

The statistical difference in the data of the various treatments was analyzed by the Kruskal–Wallis test. The post hoc test for checking individual differences was Dunn’s test. The 0.05, 0.01, and 0.001 levels of probability were taken as statistically significant.

## Data Availability

All data and materials in this study are available in this published article.

## References

[1] Carrillo C, Cavia DM, Alonso-Torre S (2012). Role of oleic acid in immune system; mechanism of action; a review. Nutr Hosp.

[2] Hwang TL, Su YC, Chang HL, Leu YL, Chung PJ, Kuo LM, Chang YJ (2009). Suppression of superoxide anion and elastase release by C_18_ unsaturated fatty acids in human neutrophils. J Lipid Res.

[3] Gonçalves-de-Albuquerque CF, Medeiros-de-Moraes IM, de Jesus Oliveira FM, Burth P, Bozza PT, Faria MVC, Silva AR, de Castro-Faria-Neto HC (2016). Omega-9 oleic acid induces fatty acid oxidation and decreases organ dysfunction and mortality in experimental sepsis. PLoS ONE.

[4] Choi KO, Choe J, Suh S, Ko S (2016). Positively charged nanostructured lipid carriers and their effect on the dissolution of poorly soluble drugs. Molecules.

[5] Natarajan JV, Nugraha C, Ng XW, Venkatraman S (2014). Sustained-release from nanocarriers: a review. J Control Release.

[6] Lin MH, Lin CF, Yang SC, Hung CF, Fang JY (2018). The interplay between nanoparticles and neutrophils. J Biomed Nanotechnol.

[7] Lin CY, Hsu CY, Elzoghby AO, Alalaiwe A, Hwang TL, Fang JY (2019). Oleic acid as the active agent and lipid matrix in cilomilast-loaded nanocarriers to assist PDE4 inhibition of activated neutrophils for mitigating psoriasis-like lesions. Acta Biomater.

[8] Bloemen M, Brullot W, Luong TT, Geukens N, Gils A, Verbiest T (2012). Improved functionalization of oleic acid-coated iron oxide nanoparticles for biomedical applications. J Nanopart Res.

[9] Tran PHL, Tran TTD, Lee BJ (2013). Enhanced solubility and modified release of poorly water-soluble drugs via self-assembled gelatin-oleic acid nanoparticles. Int J Pharm.

[10] Shah K, Chan LW, Wong TW (2017). Critical physicochemical and biological attributes of nanoemulsions for pulmonary delivery of rifampicin by nebulization technique in tuberculosis treatment. Drug Deliv..

[11] Hickey JW, Santos JL, Williford JM, Mao HQ (2015). Control of polymeric nanoparticle size to improve therapeutic delivery. J Control Release.

[12] Butt Y, Kurdowska A, Allen TC (2016). Acute lung injury: a clinical and molecular review. Arch Pathol Lab Med.

[13] Potey PMD, Rossi AG, Lucas CD, Dorward DA (2019). Neutrophils in the initiation and resolution of acute pulmonary inflammation: understanding biological function and therapeutic potential. J Pathol..

[14] Weber S, Zimmer A, Pardeike J (2014). Solid lipid nanoparticles (SLN) and nanostructured lipid carriers (NLC) for pulmonary application: a review of the state of the art. Eur J Pharm Biopharm.

[15] Hough CL (2014). Should we ever give steroids to ARDS patients?. Clin Chest Med.

[16] Fang JY, Lin YK, Wang PW, Alalaiwe A, Yang YC, Yang SC (2019). The droplet-size effect of squalene@cetylpyridinium chloride nanoemulsions on antimicrobial potency against planktonic and biofilm MRSA. Int J Nanomed.

[17] Zhao Z, Ukidve A, Krishnan V, Mitragotri S (2019). Effect of physicochemical and surface properties on in vivo fate of drug nanocarriers. Adv Drug Deliv Rev.

[18] Fromen CA, Rahhal TB, Robbins GR, Kai MP, Shen TW, Luft JC, DeSimine JM (2016). Nanoparticle surface charge impacts distribution, uptake and lymph node trafficking by pulmonary antigen-presenting cells. Nanomed Nanotechnol Biol Med.

[19] Suk JS, Kim AJ, Trehan K, Schneider CS, Cebotaru L, Woodward OM, Boylan NJ, Boyle MP, Lai SK, Guggino WB, Hanes J (2014). Lung gene therapy with highly compacted DNA nanoparticles that overcome the mucus barrier. J Control Release.

[20] Song JH, Kim JY, Piao C, Lee S, Kim B, Song SJ, Choi JS, Lee M (2016). Delivery of the high mobility group box 1 box A peptide using heparin in the acute lung injury animal models. J Control Release.

[21] Schütz C, Staedler D, Crosbie-Staunton K, Movia D, Bernasconi CC, Kenzaoui BH, Prina-Mello A, Juillerat-Jeanneret L (2014). Differential stress reaction of human colon cells to oleic-acid-stabilized and unstabilized ultrasmall iron oxide nanoparticles. Int J Nanomed.

[22] Fang B, Zhang M, Wu H, Fan X, Ren F (2017). Internalization properties of the anti-tumor α-lactalbumin-oleic acid complex. Int J Biol Macromol.

[23] Padovese R, Curi R (2009). Modulation of rat neutrophil function in vitro by cis- and trans-MUFA. Br J Nutr.

[24] Anselmo AC, Mitragotri S (2017). Impact of particle elasticity on particle-based drug delivery systems. Adv Drug Deliv Rev.

[25] Boraschi D, Italiani P, Palomba R, Decuzzi P, Duschl A, Fadeel B, Moghimi SM (2017). Nanoparticles and innate immunity: new perspectives on host defense. Semin Immunol.

[26] Hoshyar N, Gray S, Han H, Bao G (2016). The effect of nanoparticle size on in vivo pharmacokinetics and cellular interaction. Nanomedicine..

[27] He C, Hu Y, Yin L, Tang C, Yin C (2010). Effects of particle size and surface charge on cellular uptake and biodistribution of polymeric nanoparticles. Biomaterials.

[28] Wang B, He X, Zhang Z, Zhao Y, Feng W (2013). Metabolism of nanomaterials in vivo: blood circulation and organ clearance. Acc Chem Res.

[29] Kelley WJ, Fromen CA, Lopez-Cazares G, Eniola-Adefeso O (2018). PEGylation of model drug carriers enhances phagocytosis by primary human neutrophils. Acta Biomater.

[30] Gifford G, Vu VP, Banda NK, Holers VM, Wang G, Groman EV, Backos D, Scheinman R, Moghimi SM, Simberg D (2019). Complement therapeutics meets nanomedicine: overcoming human complement activation and leukocyte uptake of nanomedicines with soluble domains of CD55. J Control Release.

[31] Alexis F, Pridgen E, Molnar LK, Farokhzad OC (2008). Factors affecting the clearance and biodistribution of polymeric nanoparticles. Mol Pharm.

[32] Gamberucci A, Fulceri R, Benedetti A (1997). Inhibition of store-dependent capacitative Ca^2+^ influx by unsaturated fatty acids. Cell Calcium.

[33] Grommes J, Soehnlein O (2011). Contribution of neutrophils to acute lung injury. Mol Med.

[34] Twaddell SH, Baines KJ, Grainge C, Gibson PG (2019). The emerging role of neutrophil extracellular traps in respiratory disease. Chest.

[35] Lefrançais E, Mallavia B, Zhuo H, Calfee CS, Looney MR (2018). Maladaptive role of neutrophil extracellular traps in pathogen-induced lung injury. JCI Insight..

[36] Hwang TL, Aljuffali IA, Hung CF, Chen CH, Fang JY (2015). The impact of cationic solid lipid nanoparticles on human neutrophil activation and formation of neutrophil extracellular traps (NETs). Chem-Biol Interact..

[37] Liz R, Simard JC, Leonardi LB, Girard D (2015). Silver nanoparticles rapidly induce atypical human neutrophil cell death by a process involving inflammatory caspases and reactive oxygen species and induce neutrophil extracellular traps release upon cell adhesion. Int Immunopharmacol.

[38] Muñoz LE, Bilyy R, Biermann MH, Kienhöfer D, Maueröder C, Hahn J, Brauner JM, Weidner D, Chen J, Scharin-Mehlmann M, Janko C, Friedrich RP, Mielenz D, Dumych T, Lootsik MD, Schauer C, Schett G, Hoffmann M, Zhao Y, Herrmann M (2016). Nanoparticles size-dependently initiate self-limiting NETosis-driven inflammation. Proc Natl Acad Sci U S A..

[39] Mukherjee SP, Lazzaretto B, Hultenby K, Newman L, Rodrigues AF, Lozano N, Kostarelos K, Malmberg P, Fadeel B (2018). Graphene oxide elicits membrane lipid change and neutrophil extracellular trap formation. Chem..

[40] Ravindran M, Khan MA, Pananiyar N (2019). Neutrophil extracellular trap formation: physiology, pathology and pharmacology. Biomolecules..

[41] Khan MA, Pace-Asciak C, Al-Hassan JM, Afzal M, Liu YF, Oommen S, Paul BM, Nair D, Palaniyar N (2018). Furanoid F-acid F6 uniquely induces NETosis compared to C16 and C18 fatty acids in human neutrophils. Biomolecules..

[42] De Backer L, Cerrada A, Pérez-Gil J, De Smedt SC, Raemdonck K (2015). Bio-inspired materials in drug delivery: exploring the role of pulmonary surfactant in siRNA inhalation therapy. J Control Release.

[43] Hidalgo A, Cruz A, Pérez-Gil J (2015). Barrier or carrier? Pulmonary surfactant and drug delivery. Eur J Pharm Biopharm.

[44] Hsu CY, Sung CT, Aljuffali IA, Chen CH, Hu KY, Fang JY (2018). Intravenous anti-MRSA phosphatiosomes mediate enhanced affinity to pulmonary surfactants for effective treatment of infectious pneumonia. Nanomed Nanotechnol Biol Med.

[45] Javidi J, Haeri A, Nowroozi F, Dadashzadeh S (2019). Pharmacokinetics, tissue distribution and excretion of Ag_2_S quantum dots in mice and rats: the effects of injection dose, particle size and surface charge. Pharm Res.

[46] D’Almeida APL, de Oliveira MTP, de Souza ÉT, de Sá Coutinho D, Ciambarella BT, Gomes CR, Terroso T, Guterres SS, Pohlmann AR, Silva PMR, Martins MA, Bernardi A (2017). α-bisabolol-loaded lipid-core nanocapsules reduce lipopolysaccharide-induced pulmonary inflammation in mice. Int J Nanomed.

[47] Xue W, Liu Y, Zhang N, Yao Y, Ma P, Wen H, Huang S, Luo Y, Fan H (2018). Effects of core size and PEG coating layer of iron oxide nanoparticles on the distribution and metabolism in mice. Int J Nanomed.

[48] Hu G, Jiao B, Shi X, Valle RP, Fan Q, Zuo YY (2013). Physicochemical properties of nanoparticles regulate translocation across pulmonary surfactant monolayer and formation of lipoprotein corona. ACS Nano.

[49] Longmire M, Choyke PL, Kobayashi H (2018). Clearance properties of nanosized particles and molecules as imaging agents: considerations and caveats. Nanomedicine..

[50] Poon W, Zhang YN, Ouyang B, Kingston BR, Wu JLY, Wilhelm S, Chan WCW (2019). Elimination pathways of nanoparticles. ACS Nano.

[51] Qin M, Qiu Z (2019). Changes in TNF-α, IL-6, IL-10 and VEGF in rats with ARDS and the effects of dexamethasone. Exp Ther Med..

[52] Xu X, Zhu Q, Zhang R, Wang Y, Niu F, Wang W, Sun D, Wang A (2017). ITRAQ-based proteomics analysis of acute lung injury induced by oleic acid in mice. Cell Physiol Biochem.

[53] Shaver CM, Bastarache JA (2014). Clinical and biological heterogeneity in ARDS: direct versus indirect lung injury. Clin Chest Med.

[54] Caudrillier A, Kessenbrock K, Gilliss BM, Nguyen JX, Marques MMB, Monestier M, Toy P, Werb Z, Looney MR (2012). Platelets induce neutrophil extracellular traps in transfusion-related acute lung injury. J Clin Invest..

[55] Farrera C, Fadeel B (2013). Macrophage clearance of neutrophil extracellular traps is a silent process. J Immunol..

[56] Gonçalves-de-Albuquerque CF, Silva AR, Burth P, Castro-Faria MV, Castro-Faria-Neto HC (2015). Acute respiratory distress syndrome: role of oleic acid-triggered lung injury and inflammation. Mediators Inflamm.

[57] Rodrigues HG, Vinolo MA, Magdalon MA, Fujiwara J, Cavalcanti H, Farsky DM, Calder SH, Hatanaka PC, Curi E (2010). Dietary free oleic and linoleic acid enhances neutrophil function and modulates the inflammatory response in rats. Lipids.

[58] Pan TL, Wang PW, Hung CF, Aljuffali IA, Dai YS, Fang JY (2016). The impact of retinol loading and surface charge on the hepatic delivery of lipid nanoparticles. Colloids Surf B.

[59] Yu HP, Hsieh PW, Chang YJ, Chung PJ, Kuo LM, Hwang TL (2011). 2-(2-Fluorobenzamido)benzoate ethyl ester (EFB-1) inhibits superoxide production by human neutrophils and attenuates hemorrhagic shock-induced organ dysfunction in rats. Free Radic Biol Med..

[60] Hsieh PW, Yu HP, Chang YJ, Hwang TL (2010). Synthesis and evaluation of benzoxazinone derivatives on activity of human neutrophil elastase and on hemorrhagic shock-induced lung injury in rats. Eur J Med Chem.

[61] Yang SC, Chen PJ, Chang SH, Weng YT, Chang FR, Chang KY, Chen CY, Kao TZ, Hwang TL (2018). Luteolin attenuates neutrophilic oxidative stress and inflammatory arthritis by inhibiting Raf1 activity. Biochem Pharmacol.

[62] Asti C, Ruggieri V, Porzio S (2000). Lipopolysaccharide-induced lung injury in mice. I. Concomitant evaluation of inflammatory cells and haemorrhagic lung damage. Pulm Pharmacol Ther..

[63] Tsai YF, Yu HP, Chung PJ, Leu YL, Kuo LM, Chen CY, Hwang TL (2015). Osthol attenuates neutrophilic oxidative stress and hemorrhagic shock-induced lung injury in rats. Biochem Pharmacol.

